# Enduring neurobehavioral effects induced by microbiota depletion during the adolescent period

**DOI:** 10.1038/s41398-020-01073-0

**Published:** 2020-11-06

**Authors:** Gilliard Lach, Christine Fülling, Thomaz F. S. Bastiaanssen, Fiona Fouhy, Aoife N. O’ Donovan, Ana Paula Ventura-Silva, Catherine Stanton, Timothy G. Dinan, John F. Cryan

**Affiliations:** 1 APC Microbiome Ireland, University College Cork, Cork, Ireland; 2 Department of Anatomy and Neuroscience, University College Cork, Cork, Ireland; 3 Teagasc Food Research Centre, Food Biosciences Department, Moorepark, Fermoy, Ireland; 4 School of Microbiology, University College Cork, Cork, Ireland; 5 Department of Psychiatry and Neurobehavioural Sciences, University College Cork, Cork, Ireland; 6 Present Address: University of Edinburgh, Edinburgh, Scotland UK

**Keywords:** Psychiatric disorders, Neuroscience, Physiology

## Abstract

The gut microbiota is an essential regulator of many aspects of host physiology. Disruption of gut microbial communities affects gut-brain communication which ultimately can manifest as changes in brain function and behaviour. Transient changes in gut microbial composition can be induced by various intrinsic and extrinsic factors, however, it is possible that enduring shifts in the microbiota composition can be achieved by perturbation at a timepoint when the gut microbiota has not fully matured or is generally unstable, such as during early life or ageing. In this study, we investigated the effects of 3-week microbiota depletion with antibiotic treatment during the adolescent period and in adulthood. Following a washout period to restore the gut microbiota, behavioural and molecular hallmarks of gut-brain communication were investigated. Our data revealed that transient microbiota depletion had long-lasting effects on microbiota composition and increased anxiety-like behaviour in mice exposed to antibiotic treatment during adolescence but not in adulthood. Similarly, gene expression in the amygdala was more severely affected in mice treated during adolescence. Taken together these data highlight the vulnerability of the gut microbiota during the critical adolescent period and the long-lasting impact manipulations of the microbiota can have on gene expression and behaviour in adulthood.

## Introduction

The adolescent period is a key developmental period which marks the transition from childhood to adulthood. It is during this last developmental stage before adulthood that the brain is highly responsive to certain environmental cues that will shape neuronal architecture and promote maturation of social behaviours, emotional and cognitive capabilities and is hence a vulnerable period for the onset of psychiatric diseases^1^. The gut microbiota composition of an adolescent is usually simpler and more unstable when compared with that of an adult, which is highly diverse and stable^2,3^. These differences are probably due to relative immaturity of the gut microbiota during the adolescent period, which makes it more vulnerable to environmental stressors such as infection, use of antibiotic and poor diet. In addition to this, gonadal hormones are peaking during the puberty and it has been shown to have a long-term on the microbiota diversity^1,4^. Overt changes in the gut microbiota composition might therefore contribute to the onset of such disease and could be targeted by the use of biotherapeutics, antibiotics or different types of diet^2,5,6^. Nonetheless, the consequences of gut microbiota manipulation during adolescence is yet to be fully understood.

The gastrointestinal tract is colonised by trillions of bacteria that are tightly associated with host physiology. When the equilibrium of the microbial milieu in the gut is shifted it can have long-lasting effects on whole-body health including the brain and behaviour^7–11^. In mammals, the initial microbiota is obtained during the birthing process and develops alongside its host from a rather instable to a highly stable and diverse community in adulthood^2,12^. Microbiota composition during the developmental period is shaped by a combination of genetic and environmental factors to be highly adapted to the host and the host’s environment^12^. However, maladaptation of the gut microbiota could affect key innate and adaptative immune signalling within the intestine and at sites anatomically remote such as the brain resulting in altered host response to infection and vaccination as well as increase the susceptibility to brain disorders^2,13^. For example, altered gut microbiota composition can immediately affect brain function by impacting the turnover and release of neurotransmitters, hormones as well as growth factors and consequently affect behavioural parameters and thereby increase the susceptibility to develop neuropsychiatric disorders^5,14–17^.

One way to study the perturbation of the gut microbiota during critical periods is by using antibiotic-induced depletion. Antibiotics are one of the most important factors influencing the gut microbiota composition and structure. Studies in both female and male mice and rats^16,18–20^ and humans^21,22^ have shown that antibiotic administration can induce changes in physiology, brain and behaviour. Antibiotic depletion of the microbiota for a defined time period represents an advantage in comparison to classic approach such as germ-free (GF) animals as the effects of microbiota depletion during the early developmental period can be avoided^23,24^. It has been shown, that the use of antibiotics to chronically deplete the intestinal microbiota during adulthood has been associated with hormonal changes and alterations in gene expression, decreased adult hippocampal neurogenesis, and changes anxiety-related responses, exploratory behaviour, and cognitive abilities^20,25,26^. Moreover, early life exposure to antibiotics induces long-lasting increases in visceral pain responses^27^ as well as altered metabolic programming^18^. We have previously shown that microbiota depletion with antibiotics commencing in adolescence all the way through adulthood resulted in deficits in anxiety and cognitive behaviour^25^. It is not clear whether such changes are a result of microbiota changes specifically in adolescence, in adulthood or a combination.

Thus, in these experiments, we investigated the consequences of gut microbiota depletion specifically during adolescence or adulthood and their associated long-term effects on emotional and cognitive behaviours and related-neurochemical measures. The use of ABX allows us to ask specific questions that are unattainable using GF mice. Moreover, as ABX usage can be high in adolescence (e.g. in acne management)^28^ it allows us to have a more translationally relevant manipulation than GF animals.

## Experimental procedure

### Animals and experimental design

Adolescent and adult male C57Bl/6OlaHsd mice (Envigo, UK) were housed 4 per cage in standard cages. All mice were housed in our animal facility and maintained under a 12-h light/dark cycle. All experiments were conducted in accordance with the European Directive 86/609/EEC. Approval by the Animal Experimentation Ethics Committee of University College Cork (2012 #45) and Health Products Regulatory Authority were obtained before commencement of all experiments. To comply with 3Rs (reduction, refinement and replacement) and animal welfare, the adolescent aspect of the experiment was run simultaneously with another experiment investigating the gut microbiota^29^. The same control group was used in this study.

**Fig. 1:**
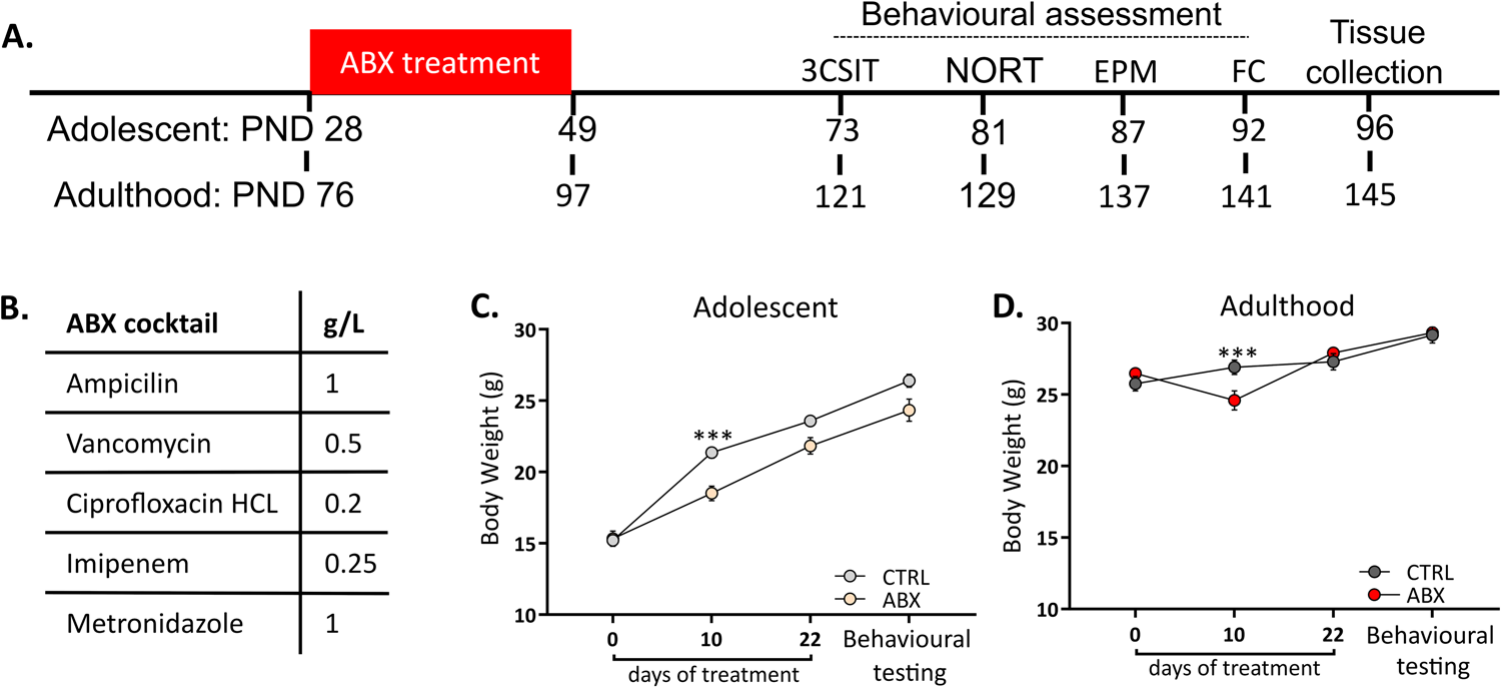
Experimental design and body weight performance during the experiment. **A** Schematic representation of the experimental timeline. Numbers represent the age of the mice at that specific timepoint. **B** List of drugs used for the antibiotic cocktail. **C**, **D** Changes in body weight during adolescence (**C**) and adulthood (**D**) over the time course of the experiment. ABX-treated mice show a significant body weight loss on PND10 which is restored afterwards. Mean ± SEM. *p < 0.05. Sample size for adolescence: CTRL *n* = 12 and ABX *n* = 10, adults: CTRL and ABX *n* = 11. Two-way repeated measures ANOVA followed by Sidak’s post hoc test. PND: postnatal day, CTRL: control, ABX: antibiotic, 3CSIT: three-chambered social interaction test, NORT: novel object recognition task, EPM: elevated plus maze, FC: fear conditioning, Beh: behavioural test.

In order to sufficiently deplete the gut microbiota, a wide-spectrum antibiotic cocktail (ABX) consisting of ampicillin (1 g/L, CAS no. 69-52-3), vancomycin (0.5 g/L, CAS no. 1404-93-9), ciprofloxacin HCL (0.2 g/L, CAS no. 93107-08-5), imipenem (0.25 g/L, CAS no. 74431-23-5) and metronidazole (1 g/L, CAS no. 443-48-1) was prepared (Fig. 1B)^20,29^. All substances were purchased from Discovery Fine Chemicals, UK. This antibiotic cocktail has little to no oral bioavailability and was prepared freshly with autoclaved water every second day for 3 weeks (Fröhlich et al., 2016). Control mice received autoclaved water (CTRL). Mice were treated during adolescence (P28-P49) or adulthood (P76-97) and will be referred to as ABX_adolescence_ and ABX_adulthood_, respectively (see Fig. 1A). Adolescence period in mice was defined as from the day where mice become sexually active (P28)^30^. Behavioural tests commenced 24 days after the final antibiotic exposure. Mice were equally assigned to experimental groups based on body weight to ensure equally distribution among the groups. Each cage represented one treatment. Behavioural tests investigating aspects of anxiety, cognition, social behaviour and fear conditioning were chosen as these behaviours have been shown to be affected by alterations in gut microbiota composition and structure^8,31–33^. Tissue samples were collected 24 h after the last behavioural test. Body weight was monitored throughout the experiment. The investigators who were involved in sample processing and data analysis were blinded to the groups. All behaviours were assessed by two independent scorers blind to the groups. See supplemental methods for detailed information of all procedures and analysis run in this study.

### Elevated plus maze

The elevated plus maze (EPM) was used to investigate anxiety-like behaviours^29^. Mice were allowed to explore the maze for 5 min; the time spent in the open arms, as well as the number of entries into the arms and head dips, were analysed.

### Novel object recognition task

Novel object recognition task (NORT) is a test for short-term memory^25^. Mice were exposed to two identical objects which they could explore for 10 min. One hour later they were exposed to a familiar and a new object. The time they spent exploring the new object was taken as an indication of their memory function.

### Three-chamber social approach test

Sociability and social novelty were investigated using the three-chamber social interaction test (3CSIT)^29^. The test consisted of three sequential 10-min trials: (1) habituation, (2) sociability, measured as the time the mouse spent in proximity to a conspecific or an object and (3) social novelty preference as measured by the time the mouse spent with an unfamiliar conspecific or a familiar one.

### Differential fear-conditioning paradigm

Fear conditioning (FC) is based on pairing an initially neutral and non-aversive stimulus, an auditory cue or context (conditioned stimulus, CS), with an aversive stimulus, such as a foot shock (unconditioned stimulus, US), which will result in a fear response in the presence of the CS^34^. The paradigm was run over 4 consecutive days: day 1 (context A): conditioning, day 2 (context A): contextual extinction, day 3 (context B): conditioned extinction in a novel context, day 4 (context B): context recall.

### RNA extractions, reverse transcription and quantitative RT-PCR

Whole amygdala and prefrontal cortex were rapidly gross-dissected on an ice-cooled Petri dish following coordinates described in the “The Mouse Brain in Stereotaxic Coordinates”^35^ and snap-frozen on dry-ice. For consistency, the same experienced researcher was responsible for all the dissection. These brain regions were chosen as they are major contributor to anxiety and fear learning (amygdala) and proper neuronal communication during development (prefrontal cortex)^36–41^. The list of genes was elaborated together with the experimental design to assess major components modulated by the gut microbiota, such as immune and microglia-related markers, tight-junction proteins and myelin- and stress-related genes^8,29,32,39,40^. In addition, based on the behavioural phenotype, we included genes associated with anxiety-like behaviour such as the genes involved in the GABAergic and glutamatergic system, NPY system and genes related to neuroplasticity^8,26,29,33,36^. Primer sequences are listed on the Supplementary Table S1. Total RNA was extracted with the mirVana total RNA extraction kit (Ambion, UK) and RNA was reverse transcribed using a high-capacity cDNA reverse transcription kit (Thermo Fisher Scientific, Waltham, USA) in a G-storm thermocycler (G-storm, Surrey, UK). Real-time PCR was performed on the cDNA samples using SYBR green (SensiFAST™ SYBR®, BioLine, UK) and gene expression levels were analysed on an AB7300 system (Applied Biosystems, Thermo Fisher Scientific, USA). Expression levels were calculated as the average of three replicates for each biological sample from all three groups relative to the endogenous control. Fold changes were calculated using the ΔΔCt method^29^. The expression of the housekeeper ACTB was not affected by ABX treatment or age.

### Caecal microbiota composition (16S rRNA gene sequecing)

The QIAmp Fast DNA Stool Mini Kit (Qiagen, Sussex, UK) was used for caecal DNA extraction. The procedure was coupled with an initial bead-beating step. Amplification and preparation for sequencing of the V3-V4 hypervariable region of the 16S rRNA gene were done as outlined in the Illumina 16S Metagenomic Sequencing Library Protocol and as previously described^29^. Briefly, microbial genomic DNA was run with each primer (forward primer (5′-TCGTCGGCAGCGTCAGATGTGTATAAGAGACAGCCTACGGGNGGCWGCAG-3′) and reverse primer (5′-GTCTCGTGGGCTCGGAGATGTGTATAAGAGACAGGACTACHVGGGTATCTAATCC-3′). PCR products were purified using the Agencourt AMPure XP system (Beckman Coulter Genomics, UK). Dual indices and Illumina sequencing adapters were attached to PCR products using the Nextera XT Index Kit (Illumina, USA). PCR products were quantified, normalized and pooled in an equimolar fashion using the Qubit® dsDNA HS Assay Kit (Life Technologies, USA). Following, samples were run on the Agilent Bioanalyser for quality analysis and samples prepared for sequencing following Illumina guidelines. Samples were sequenced on the MiSeq sequencing platform (Clinical Microbiomics, Denmark).

#### Microbiome bioinformatics processing

Three-hundred base-pair paired-end reads were prefiltered based on a quality score threshold of >28 and trimmed, filtered for quality and chimaeras using the DADA2 library in R^42^. Samples with fewer than 10,000 reads after trimming and filtering were dropped. Taxonomy was assigned with DADA2 against the SILVA SSURef database release v132. Parameters, as recommended in the DADA2 manual, were adhered to unless mentioned otherwise. ASVs that were only detected as non-zero in 2 or fewer of total samples were excluded.

### Statistics

Power analysis was performed beforehand using the Software G*Power 3.1 to ensure adequate sample size number to detect changes in behaviour and gene expression. Statistical analysis and plotting were conducted using Prism 7 (GraphPad, USA). Data were checked for normality using Shapiro–Wilks normality test while the ROUT method [50] was used to check for outliers. Two-tailed Student’s *t*-test (for equal variances) or Welch’s *t*-test (for unequal variances) were used for comparison between CTRL and ABX for the EPM, NORT, context and extinction recall in FC, and gene expression. Mann–Whitney nonparametric test was used when dataset failed to have a normal distribution. Two-way repeated measures analysis of variance (ANOVA) was used for body weight, 3CSIT, as well as acquisition and cued extinction in FC. Sidak’s multiple comparisons post hoc test was used where applicable. Statistical significance was set at *p* < 0.05.

Statistical analysis of microbiota data was performed using the R software (version 3.6) environment with Rstudio (version 1.1.453). Alpha diversity was calculated using the iNEXT library^43^. Wilcoxon rank sum followed by Bonferroni post hoc tests were used to assess differences in Alpha-diversity scores. For principal component analysis (PCA), permutational multivariate analysis of variance (PERMANOVA) was used to identify relationships of significance between variables using the adonis() function from the vegan library on Aitchison distance matrices calculated with the ALDEx2 library^44^. A pairwise implementation of the ALDEx2 function aldex.t.test() was also used to calculate pairwise differential abundance, using the Bonferroni procedure as a post hoc. In the case of microbiome analysis, the Benjamini–Hochberg procedure was used to account for false discovery rate due to multiple comparisons, a *q*-value of 0.1 was deemed significant.

## Results

### Antibiotic treatment in adolescence affects body weight

Two-way repeated measures ANOVA showed an overall effect of ABX treatment during adolescence on body weight (interaction F(3,48) = 3.84, *p* = 0.015, treatment F(1,16) = 7.18, *p* = 0.017), whereas such an effect was not seen following treatment with ABX_adulthood_ (interaction F(3,60) = 12.56, *p* < 0.001, treatment F(1,20) = 0.11, *p* = 0.75; Fig. 1C, D). Sidak’s multiple comparison, however, revealed that body weight was significantly lower in the ABX treatment in both ABX_adolescence_ and ABX_adulthood_ mice on day 10 of the treatment (*p* = 0.003 and *p* = 0.05, respectively). On day 10, the body weight loss in ABX-treated mice compared to the CTRL was 14% and 12% for adolescent and adults mice, respectively.

### Microbial diversity was only affected by ABX treatment during adolescence

**Fig. 2:**
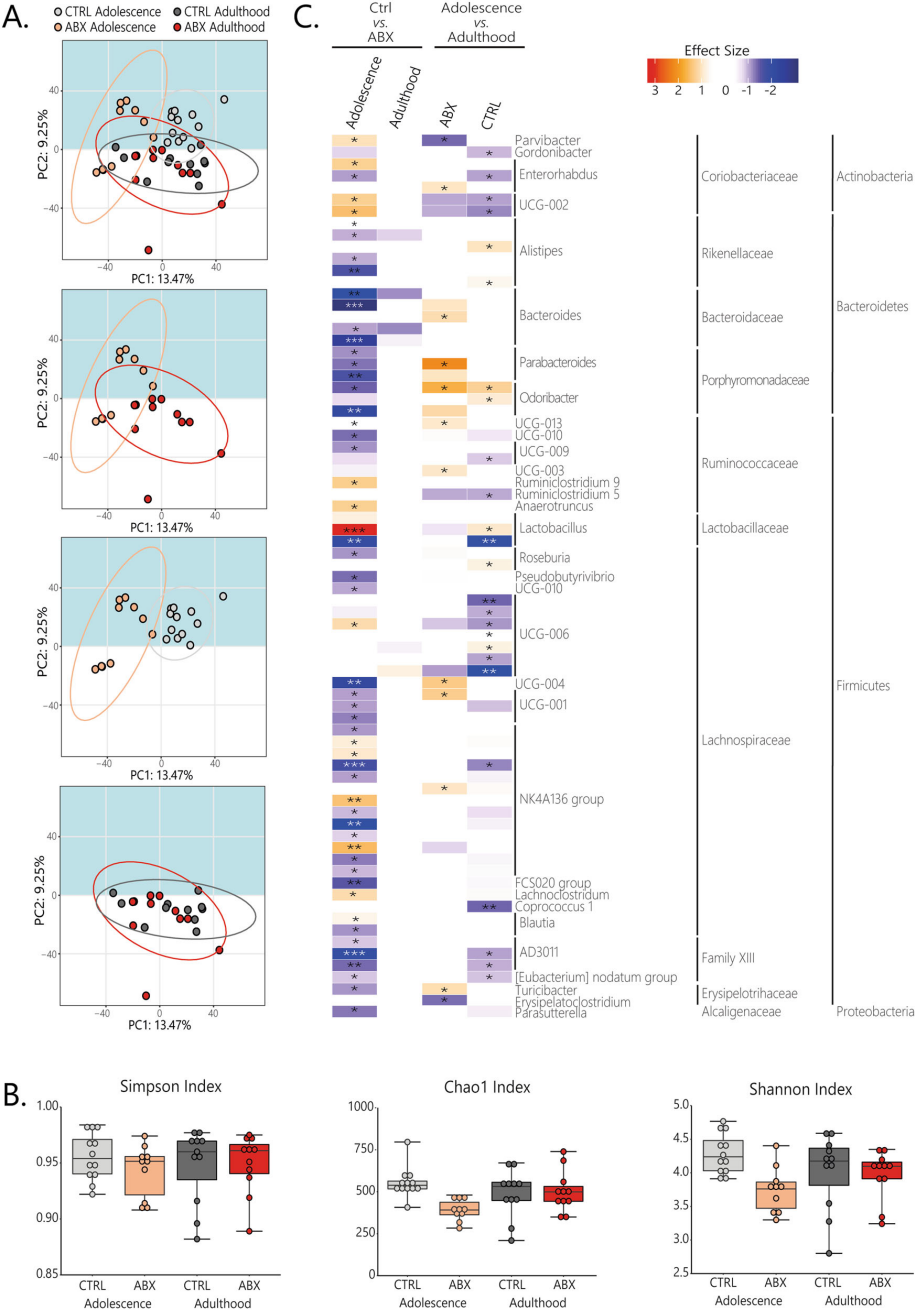
Long-lasting changes in caecal microbiota composition following ABX intervention during adolescence. **A** PCA blots depicting differences in beta-diversity between mice treated with ABX and their respective controls. While beta-diversity was affected by ABX_adolescence_ treatment no such effects were seen following ABX_adulthood_ treatment. **B** alpha-diversity indices: Chao1, Shannon and Simpson. Changes in the Chao1 and Simpson indices are observed following treatment with ABX during adolescence but not after treatment in adulthood. **C** Heat map representing relative abundance of ASVs. Significant differences were observed between control mice and ABX-treated mice during adolescence but not when treated during adulthood. Some differences in ASVs were observed when comparing both ABX treatments and controls with one another. The latter does not explain the effect on relative abundance of ABX treatment during adolescence. Mean ± SEM. *p < 0.05. Sample size for adolescence: CTRL *n* = 12 and ABX *n* = 10; for adults: CTRL and ABX *n* = 11. **A** Permutational multivariate analysis of variance (PERMANOVA), followed by pairwise PERMANOVA post hoc Benjamini–Hochberg, **B** Wilcoxon rank-sum test, post hoc Bonferroni, *p < 0.05, **C** Mann–Whitney U test post hoc Benjamini–Hochberg, Benjamini–Hochberg false discovery rate (FDR) *q* < 0.2. Asterisks in the heat map represent the following *q* values: *p < 0.1, **p < 0.01, ***p < 0.001.

To investigate possible long-lasting effects of ABX treatment during adolescence and adulthood on the gut microbiota, microbiota was sequenced from caecal contents 49 days after termination of the treatment. Overall, sequencing demonstrated that the gut microbiota was shifted only in ABX_adolescence_ mice where ABX treatment had reduced relative abundance and diversity compared to CTRL (Fig. 2A–C). With regards to beta-diversity, PERMANOVA identified significant differences between CTRL and ABX treatment in adolescent mice (*p* < 0.001, *R*^2^ = 0.193, padj = 0.004; Fig. 2A), but no such change was observed in ABX_adulthood_ (*p* = 0.029, *R*^2^ = 0.071, padj = 0.174; Fig. 2A).

Alpha diversity, which describes the diversity of species within a community, was measured by calculating indices for richness and evenness based on amplicon sequence variants (ASV) level. Details of the statistics for each ASV can be found on the Supplementary Table S2. Figure 2B shows reduced numbers of bacterial species between CTRL and ABX treatment in adolescent mice (Chao1 Index; pairwise comparison using Wilcoxon rank-sum test, *p* < 0.001) while no changes were observed in ABX_adulthood_ mice. Moreover, a significant decrease in evenness across ASVs was observed in ABX_adolescence_ mice (Shannon Index; pairwise comparison using Wilcoxon rank-sum test, *p* < 0.001), indicating that treatment with ABX during adolescence disrupted the uniformity of the population size of each of the species present. No effects of treatment during adolescence or adulthood were observed for the Simpson’s index.

ABX treatment only resulted in significant long-lasting changes in relative abundance of adolescent mice. ABX_adolescence_ mice showed reduced relative abundance of most of the strains affected. At the phylum level, Firmicutes and Bacteriodetes were most affected by the ABX_adolescence_ intervention (Fig. 2C). Actinobacteria had a marginal shift that was restricted to the genus *Coriaobacteriaceae* UCG002 and *Parvibacter*, found increased, and *Enterorhabdus*, with mixed result. Relative abundance of all the affected genera in the phylum Bacteriodetes was decreased, with the strongest reduction in the genera *Bacteroides*, while *Parabacteroides, Odoribacter* and *Alistipes* had less strong effect. Members of the Firmicutes phylum were differentially in ABX_adolescence_. In the *Lachnospiraceae* family, relative abundance of all affected ASV of the genera *Rosburia*, *UCG-001*, *UCG-004*, *UCG-010*, *FCS020 group* and *Pseudobutyrivibrio* were decreased, whereas at *UCG-006*, *NK4A136 group*, *Lachnoclostridium* and *Blautia* showed increased relative abundance in ABX_adolescence_. Of the family *Ruminococcaceae*, relative abundance of the genera *UCG-003*, *UCG-009*, *UCG-010* and *UCG-013* was decreased while the genera *Ruminiclostridium 5*, *Ruminiclostridium 9* and *Anaerotruncus* was significantly increased. Similarly, one ASV of the genus *Lactobacillus* was decreased while another ASV of this genus was strongly increased following treatment with ABX. The relative abundance of all affected ASVs of the family *Family XIII* and *Eryspelotrihaceae* was decreased. The phylum Proteobacteria had just one family changed after ABX treatment, where *Parasutterella* was found decreased.

In ABX_adulthood_, no significant effect on relative abundance was observed. However, differences in relative abundance were observed in CTRL or ABX groups between adolescent and adults mice. As expected, CTRL mice from adolescence and adults have different microbiota composition and diversity (Fig. 2A–C). When comparing ABX treatment of adolescent versus adult mice, relative abundance of most of the ASVs is increased in ABX_adulthood_ mice in comparison to ABX_adolescence_ treated mice and vice versa (e.g. ASVs of the genera *Parabacteroides* and *Odoribacter*). This is in line with the decrease observed in the comparison of relative abundance of ABX_adolescence_ mice and their controls. Similarly, the differences seen between the controls of either treatment is not in contradiction with the differences observed in relative abundance of ABX_adolescence_ mice in comparison to their controls. The differences in relative abundance between controls mostly occurred in ASVs that are not affected in the ABX_adolescence_, 14 out of the 25 ASVs that were different between the controls were not significantly affected by ABX_adolescence_ treatment (e.g. ASVs of the genus *Alistipedis*).

### Treatment with antibiotics strongly affected anxiety-like behaviour, with a more pronounced effect in mice treated during adolescence

**Fig. 3:**
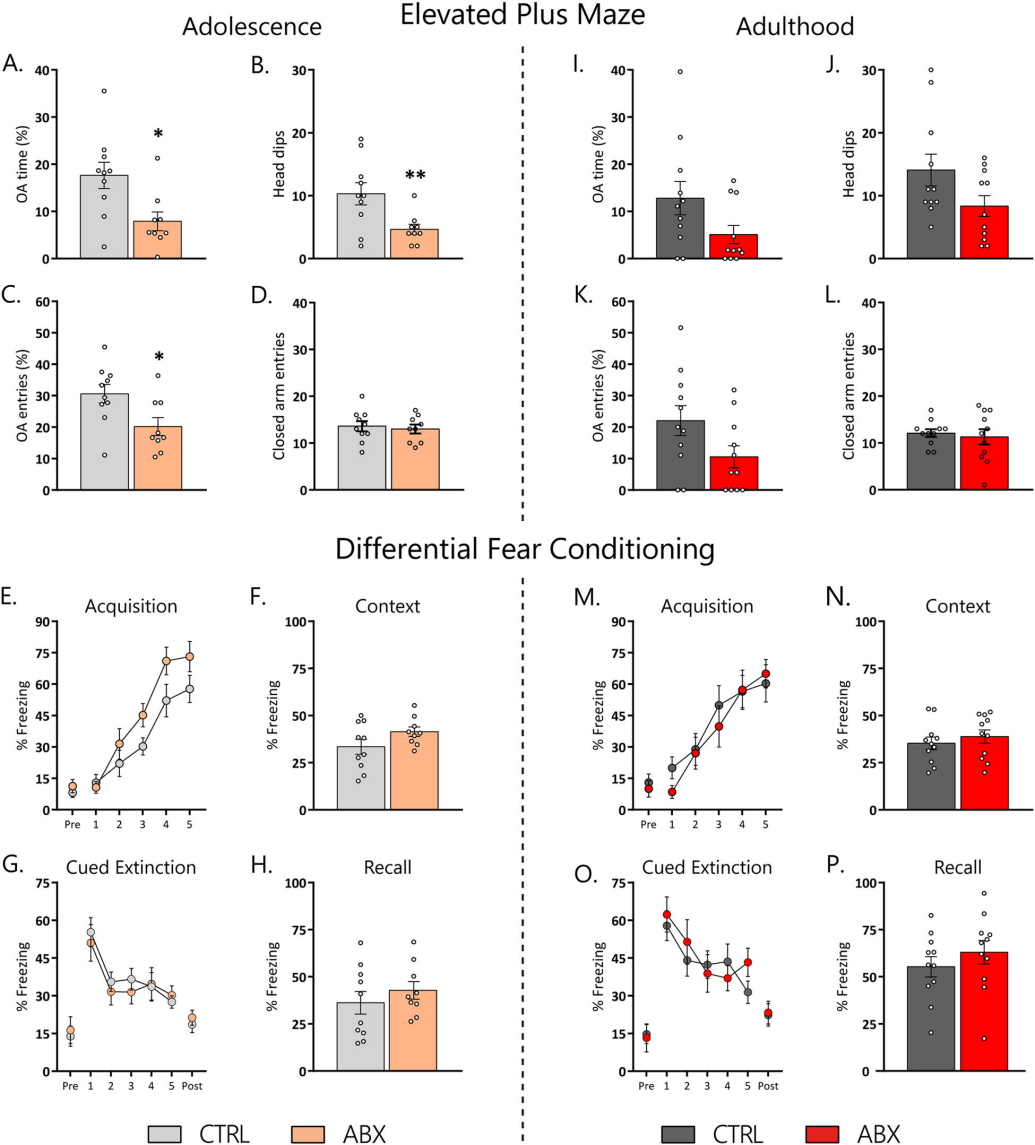
ABX treatment during adolescence affects anxiety-like behaviour and the fear response during fear acquisition. **A**–**D** Mice treated with ABX during adolescence showed increased anxiety-like behaviour as they showed decreased % time in and % entries into the open arm as well as decreased number of head dips. **I**–**L** No significant decrease in anxiety-like behaviour was seen in mice treated with ABX during adulthood. **E**–**H** An effect of treatment was seen for the acquisition of fear conditioning in mice treated with ABX during adolescence, but no other parameter was affected. **M**–**P** Treatment with ABX during adulthood had no effect on any parameter measured in the fear-conditioning paradigm. Mean ± SEM. **A**–**D**, **F**, **H**, **I**–**L** Welsh’s *t*-test comparison between CTRL and ABX. **E**, **G**, **M**, **O** two-way repeated measures ANOVA, followed by Sidak’s post hoc test when applicable *p < 0.05 and **p < 0.01. Sample size for adolescence (CTRL: *n* = 9; ABX: *n* = 10) and adulthood (CTRL: *n* = 11; ABX: *n* = 11).

To test the effects of ABX treatment on anxiety-like behaviour, mice were tested in the EPM. Mice that underwent antibiotic treatment showed increased anxiety-like behaviour. Unpaired *t*-test with Welch’s correction showed that the percentage of time into the open arms, as well as the number of head dips (*t* = 2.846, *p* = 0.01 and *t* = 2.909, *p* = 0.01, respectively; Fig. 3A, B), were affected significantly in ABX_adolescence_ mice. Student *t*-test showed that the percentage of entries into the open arms also was affected significantly in ABX_adolescence_ mice (*t* = 2.529, *p* = 0.02; Fig. 3C). Welch’s *t*-test revealed no statistical significance in the percentage of entries into the open arm in ABX_adulthood_ mice (*t* = 1.946, *p* = 0.06; Fig. 3K). Mann–Whitney nonparametric test found that the percentage of time into the open arms as well as head dips were no difference in ABX_adulthood_ compared to the controls (*U* = 38, *p* = 0.148 and *U* = 38, *p* = 0.145, respectively; Fig. 3I, J). Entries into the closed arm did not differ between treatment groups (adolescence: *t* = 0.419, *p* = 0.68; adulthood: *t* = 0.449, *p* = 0.65; Fig. 3D, L), indicating that ABX treatment did not affect locomotor activity.

### Sociability and memory processes were not affected by antibiotic treatment in adolescence or adulthood

As social behaviour and cognition have been shown to be impacted by alterations in gut microbiota composition, we investigated the effects of ABX treatment on measures in 3CSIT and NORT. ABX treatment during adolescence or adulthood had no effects on sociability or social novelty. Two-way repeated measures ANOVA found preferential interaction for a stranger mouse (S1) over an innate object (O) (S1 × O: F(1,17) = 52.92, *p* < 0.001, treatment: F(1,17) = 1.76, *p* = 0.20 for adolescence; S1 × O: F(1,20) = 60.54, *p* < 0.001, treatment: F(1,20) = 0.013, *p* = 0.90 for adulthood, supplementary Fig. 1B, E, respectively). Sidak’s multiple comparison post hoc test found that CTRL and ABX from both adolescence and adult groups preferred S1 over O (*p* < 0.0001). On social novelty, two-way repeated measures ANOVA found that only the adolescence group has a preference between stranger and familiar mouse (S1 × S2: F(1,17) = 13.96, *p* < 0.01, treatment: F(1,17) = 3.03, *p* = 0.09 for adolescence; S1 × S2: F(1,20) = 2.74, *p* = 0.11, treatment: F(1,20) = 0.61, *p* = 0.44 for adulthood, supplementary Fig. 1C, F, respectively). Sidak’s multiple comparison post hoc test found that ABX-treated adolescent preferred S1 over S2 (*p* < 0.001) but not CTRL-treated mice (*p* = 0.10).

In the NORT, neither ABX_adolescence_ or ABX_adulthood_ exhibited differences in the ability to recognize a distinct object 1 h after the training session, expressed by the time exploring the novel object (adolescence: *t* = 1.233, df = 16.47, *p* = 0.23; adulthood: *t* = 0.5288, df = 16.77, *p* = 0.60, Supplementary Fig. 2A, D).

### Fear learning is only affected in animals treated with antibiotics during adolescence

Fear conditioning aimed to investigate different fear responses, such as fear learning, contextual fear and cued extinction (Fig. 3E–H, M–P). All experimental groups learned the task as indicated by increasing freezing behaviour during acquisition (two-way repeated measures ANOVA; CS presentation, adolescence: F(4,68) = 38.11, *p* < 0.001; adulthood: F(4,80) = 27.36, *p* < 0.001; Fig. 3E, M). Only ABX_adolescence_ mice significantly increased freezing behaviour compared to the control group during fear acquisition, while in adults, ABX did not affect fear acquisition (two-way repeated measures ANOVA; treatment, adolescence: F(1,17) = 4.48, *p* = 0.04; adulthood: F(1,20) = 0.17, *p* = 0.67). Sidak’s multiple comparison post hoc test did not find a significance between CTRL and ABX in any timepoint in both adolescence and adults mice. On day 2, contextual fear was assessed. Unpaired *t*-test with Welch’s correction revealed that the treatment did not affect freezing behaviour in adolescent mice (*t* = 1.70, *p* = 0.10; Fig. 3F). Student *t*-test revealed the same in adult mice (*t* = 0.73, *p* = 0.47; Fig. 3N). In the cued extinction session (day 3), the response to the CS (paired sound) was analysed. All experimental groups reduced their freezing response over the course of CS presentation (CS presentation, two-way repeated measures ANOVA adolescence: F(4,68) = 18.03, *p* < 0.001, adulthood: F(4,80) = 15.15, *p* < 0.001; Fig. 3G, O), suggesting that all experimental groups were able to extinguish cued fear. Fear extinction was not affected by treatment (two-way repeated measures ANOVA; adolescence: F(1,17) = 0.095, *p* = 0.76, adulthood: F(1,20) = 0.11, *p* = 0.74). Pre and post CS freezing were similar in ABX-treated mice compared to their controls (Welch’s *t*-test; adolescence; pre: *t* = 0.36, *p* = 0.72; post: *t* = 0.61, *p* = 0.54; adulthood; pre: *t* = 0.22, *p* = 0.82; post: *t* = 0.15, *p* = 0.88), indicating the absence of generalized fear. Fear recall was tested on day four and no difference was observed (Welch’s *t*-test; adolescence: *t* = 0.86, *p* = 0.39, adulthood *t* = 0.93, *p* = 0.36; Fig. 3H, P). Taken together, this data show that transient depletion of the gut microbiota has little potential to long-lasting affect fear response.

### Amygdala gene expression is more strongly affected by treatment during adolescence than adulthood

**Fig. 4:**
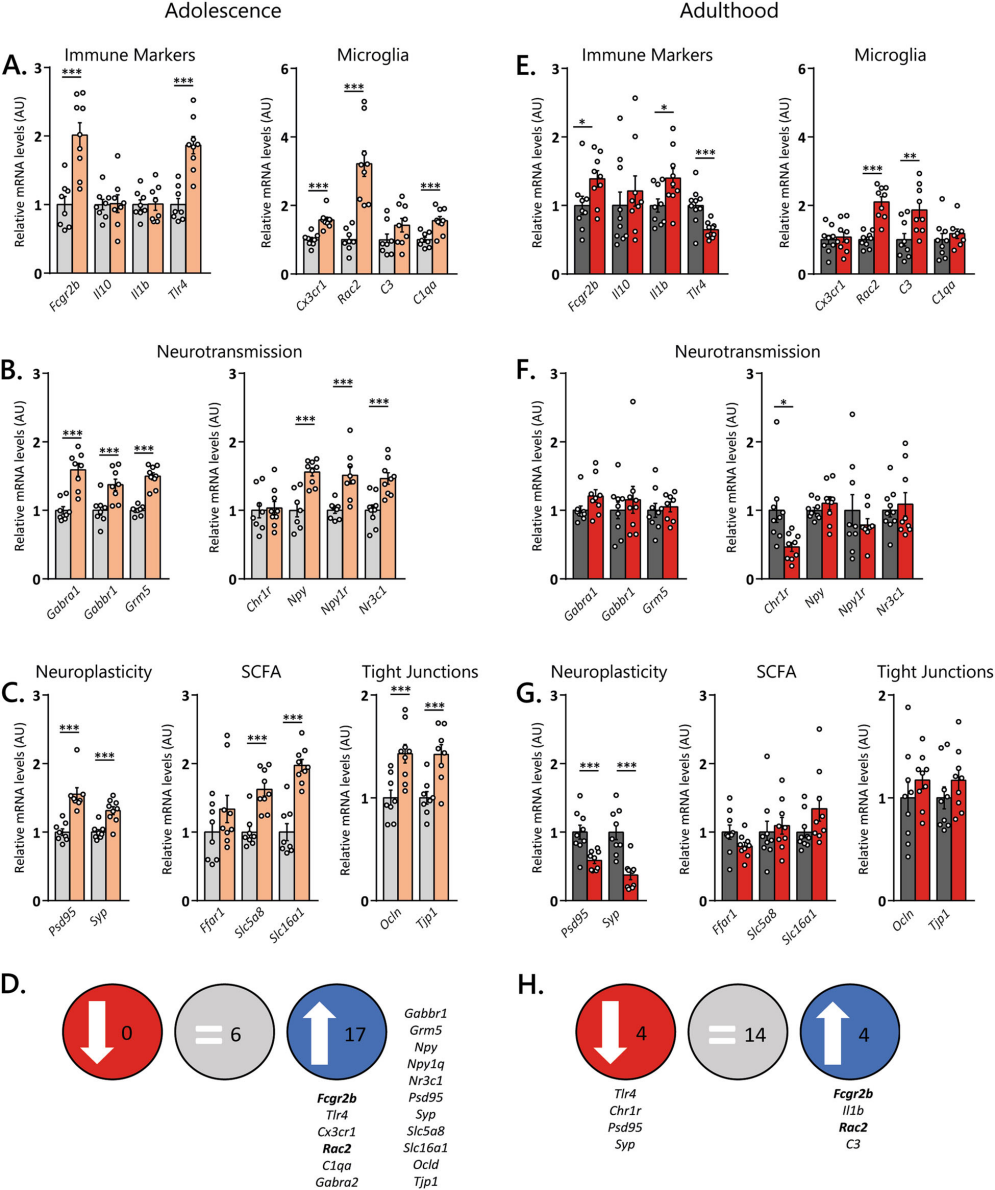
Amygdalar gene expression is more drastically changed following ABX treatment during adolescence. **A**, **B** While genes related to neuroimmunity and microglia are similarly affected in ABX_adolescence_ and ABX_adulthood_ mice, gene expression genes involved in **C**, **D** neurotransmission, **E**, **F** neuroplasticity, short-chain fatty acids and tight-junction proteins are more drastically affected in ABX_adolescence_ mice. Mean ± SEM. Unpaired *t*-test with Welch’s correction comparing vehicle and antibiotic treatment for each gene. *p < 0.05, **p < 0.01 and ***p < 0.001. Sample size for adolescence: CTRL and ABX *n* = 8 (*Il10*, *Il1b*, *Gabra2*, *Gabbr1*, *Npy1r*, *Psd95* and *Tjp1*), CTRL *n* = 8 and ABX *n* = 9 (*Fcgr2b*, *Cx3cr1*, *Rac2*, *C3*, *C1qa*, *Grm5*, *Chr1r*, *Npy*, *Syp*, *Ffar1*, *Slc5a8*, *Slc16a1*, *Ocln*), CTRL *n* = 9 and ABX *n* = 8 (*Tlr4*), CTRL and ABX *n* = 9 (*Nr3c1*). Sample size for adults: CTRL and ABX *n* = 9 (*Fcgr2b*, *Il1b*, *Cx3cr1*, *Rac2*, *C3*, *Gabra2*, *Gabbr1*, *Grm5*, *Crh1r*, *Npy1r*, *Psd95*, *Syp*, *Ffar1*, *Slc5a8*, *Ocln, Tjp1*), CTRL *n* = 10 and ABX *n* = 9 (*Il10*, *Tlr4*, *Nr3c1*, *Slc16a1*), CRTL *n* = 9 and ABX *n* = 8 (*C1qa*, *Npy*). SCFA: short-chain fatty acid, *C1qa*: complement C1q subunit A, *C3*: complement component 3, *Crh1r*: corticotropin-releasing hormone receptor 1, *Cx3cr1*: chemokine receptor 1, *Fcgr2b*: Fc fragment of IgG receptor IIb, *Ffar1*: free fatty acid receptor 1, *Gabrb1*: gamma-aminobutyric acid type B receptor subunit 1, *Gabra2*: gamma-aminobutyric acid type A receptor alpha2 subunit, *Grm5*: glutamate metabotropic receptor 5, *Il1b*: interleukin 1b, *Il10*: interleukin 10, *Npy*: neuropeptide Y, *Npy1r*: neuropeptide Y receptor Y1, *Nr3c1*: glucocorticoid receptor, *Ocln*: occludin, *PSD95*: postsynaptic density protein *95, Rac2*: Ras-related C3 botulinum toxin substrate 2, *Slc5a8*: solute carrier family 5 member 8*, Slc16a1*: solute carrier family 16 member 1, *Syp*: Synaptophysin, *Tjp1*: tight-junction protein 1, *Tlr4*: toll-like receptor 4.

Many studies have demonstrated a link between the gut microbiota and gene expression in the amygdala^39,45^. In amygdala homogenates, mRNA levels revealed a stronger effect of ABX_adolescence_ than ABX_adulthood_ treatment (Fig. 4 and Table 1). Immune markers were heavily affected by ABX treatment during adolescence and adulthood (Fig. 4A, E). For instance, elevated levels of *Fcgr2b* (Fc fragment of IgG receptor IIb) gene were observed in both ABX_adolescence_ and ABX_adulthood_ mice. The toll-like receptor 4 gene (*Tlr4*) was oppositely affected by ABX treatment with elevated gene expression following ABX_adolescence_ treatment and reduced expression after ABX_adulthood_ treatment. In contrast, interleukin 1b (*Il1b*) was elevated following ABX_adulthood_ treatment but not following ABX_adolescence_ treatment, while the microglia-related gene *C1qa* (encodes the A-chain polypeptide of serum complement subcomponent C1q) and *Cx3cr1* (interleukin 8 receptor, alpha) was only upregulated in ABX_adolescence_ mice. *Rac2* (Rho GTPase Rac2), which regulates phagocytosis was upregulated in both experimental groups. The complement component 3 (*C3*) which is also involved in the regulation of phagocytosis, was only upregulated in ABX_adulthood_ mice. Classical and peptidergic neurotransmission was strongly affected by ABX_adolescence_ treatment but remained mostly unaltered in ABX_adulthood_ mice (Fig. 4B, F). The levels of gamma-aminobutyric acid receptor subunit alpha-2a (*Gabra2*) and beta-1b (*Gabrb1*), the metabotropic glutamate receptor subunit 5 (*Grm5*), neuropeptide Y (NPY) and its Y1 receptor (*Npy1r*) and the glucocorticoid receptor (*Nr3c1*) were only increased in ABX_adolescence_ mice. The corticotropin-releasing hormone receptor subunit 1 (*Crh1r*) was the only gene affected in ABX_adulthood_ mice, that was not altered by ABX_adolescence_ treatment. Neuroplasticity genes were differently affected by ABX_adolescence_ and ABX_adulthood_ (Fig. 4C, G). While the levels of postsynaptic density protein 95 (*Psd95*) and synaptophysin (*Syp*) were increased in ABX_adolescence_ mice, these genes were downregulated following ABX_adulthood_ treatment. Interestingly, genes associated with short-chain fatty acids (SCFAs), the solute carrier subtypes (*Slc5a8* and *Slc16a1*) were only elevated in ABX_adolescence_ mice. Similarly, the gene expression of tight-junction genes, such as occludin (*Ocln*) and tight-junction protein 1 (*Tjp1*) were only increased in ABX_adolescence_ mice. Taken together, although more genes are affected in ABX_adolescence_, immune-related genes are similarly affected by both ABX treatments.

**Table 1:** Statistical values of the gene expression in the amygdala, prefrontal cortex following ABX intervention during adolescence and adult.

	Adolescent		Adult	
Gene	*t*, df/U, *t*_1/2_	*p*	*t*, df/U, *t*_1/2_	*p*
*Amygdala*				
*Fcgr2b*	*t*_(W)_ = 4.732, df = 13.59	<0.001	*t*_(W)_ = 2.167, df = 15.8	0.05
*C1qa*	*t*_(W)_ = 3.938, df = 13.16	<0.001	*t*_(W)_ = 0.765, df = 14.37	0.46
*Tlr4*	*t*_(W)_ = 5.658, df = 13.92	<0.001	*U* = 10; *t*_1_ = 135, *t*_2_ = 55	0.003
*Il10*	*t*_(W)_ = 0.097, df = 11.27	0.92	*U* = 32; *t*_1_ = 87, *t*_2_ = 103	0.31
*Il1B*	*t*_(W)_ = 0.079, df = 12.96	0.94	*t*_(W)_ = 2.356, df = 14.15	0.03
*Cx3cr1*	*U* = 1; *t*_1_ = 37, *t*_2_ = 116	<0.001	*t*_(W)_ = 0.400, df = 15.72	0.69
*Rac2*	*t*_(W)_ = 5.679, df = 9.484	<0.001	*t*_(W)_ = 6.379, df = 11.64	<0.001
*C3*	*t*_(W)_ = 1.643, df = 14.89	0.12	*t*_(W)_ = 2.951, df = 15.29	0.01
*Gabra2*	*t*_(W)_ = 5.427, df = 11.06	<0.001	*U* = 20; *t*_1_ = 65, *t*_2_ = 106	0.07
*Gabrb1*	*t*_(W)_ = 3.42, df = 13.44	<0.001	*U* = 37; *t*_1_ = 82, *t*_2_ = 89	0.79
*Grm5*	*t*_(W)_ = 8.899, df = 11.21	<0.001	*t*_(W)_ = 0.409, df = 13.89	0.69
*Psd95*	*U* = 0; *t*_1_ = 36, *t*_2_ = 100	<0.001	*t*_(W)_ = 3.724, df = 11.06	<0.001
*Syp*	*t*_(W)_ = 4.609, df = 13.46	<0.001	*t*_(W)_ = 4.88, df = 13.49	<0.001
*Npy*	*t*_(W)_ = 5.087, df = 12.06	<0.001	*t*_(W)_ = 0.865, df = 10.51	0.41
*Nr3c1*	*t* = 4.087, df = 16	<0.001	*U* = 39; *t*_1_ = 106, *t*_2_ = 84	0.64
*Npy1r*	*t*_(W)_ = 3.842, df = 8.787	<0.001	*t*_(W)_ = 0.992, df = 11.31	0.34
*Chr1r*	*t* = 0.2401, df = 15	0.81	*U* = 8; *t*_1_ = 118, *t*_2_ = 53	0.02
*FFAR1*	*t*_(W)_ = 26; *t*_1_ = 62, *t*_2_ = 91	0.19	*t*_(W)_ = 1.736, df = 12.16	0.11
*Slc5a8*	*U* = 4; t_1_ = 40, *t*_2_ = 113	<0.001	*U* = 30; *t*_1_ = 75, *t*_2_ = 96	0.38
*Slc16a1*	*t*_(W)_ = 6.466, df = 12.82	<0.001	*U* = 21; *t*_1_ = 76, *t*_2_ = 114	0.05
*Ocln*	*t*_(W)_ = 3.641, df = 14.88	<0.001	0.34	
*Tjp1*	*t*_(W)_ = 3.759, df = 12.05	<0.001	*t* = 1.171, df = 16	0.26
*Prefrontal cortex*				
*Myrf*	*U* = 36; *t*_1_ = 91, *t*_2_ = 99	0.49	*t*_(W)_ = 7.641, df = 14.12	<0.001
*Plp1*	*t*_(W)_ = 0.489, df = 14.92	0.63	*U* = 45; *t*_1_ = 111, *t*_2_ = 120	0.49
*Sox10*	*t*_(W)_ = 0.762, df = 16.89	0.46	*t* = 4.934, df = 19	<0.001

Mean ± SEM. Unpaired *t*-test with or without Welch’s correction or Mann–Whitney whenever the dataset did not follow normal distribution.

*t* Student *t*-test, *t*_(W)_ Student *t*-test with Welch’s correction, *df* degree of freedom, *U* Mann–Whitney test, *t*_1/2_ sum of ranks of CTRL and ABX, *C1qa* complement C1q subunit A, *C3* complement component 3, *Crh1r* corticotropin-releasing hormone receptor 1, *Cx3cr1* chemokine receptor 1, *Fcgr2b* Fc fragment of IgG receptor IIb, *Ffar1* free fatty acid receptor 1, *Gabrb1* gamma-aminobutyric acid type B receptor subunit 1, *Gabra2* gamma-aminobutyric acid type A receptor alpha2 subunit, *Grm5* glutamate metabotropic receptor 5, *Il1b* Interleukin 1b, *Il10* interleukin 10, *Myrf* myelin regulatory factor, *Npy* neuropeptide Y, *Npy1r* neuropeptide Y receptor Y1, *Nr3c1* glucocorticoid receptor, *Ocln* occludin, *Plp1* proteolipid protein 1, *Psd95* postsynaptic density protein 95*, Rac2* Ras-related C3 botulinum toxin substrate 2*, Slc5a8* solute carrier family 5 member 8*, Slc16a1* solute carrier family 16 member 1, *Sox10* SRY-box transcription factor 10, *Syp* synaptophysin, *Tjp1* tight-junction protein 1, *Tlr4* toll-like receptor 4.

Finally, we analysed myelination-related genes in the prefrontal cortex, which have been suggested to be modulated by the gut microbiota^20,46^. Therefore, we analysed gene expression of myelin regulatory factor (*Myrf*), proteolipid protein 1 (*Plp1*) and the transcription factor SOX-10 (*Sox10*). ABX_adulthood_ treatment resulted in increased gene expression of *Myrf* and *Sox10* while ABX_adolescence_ showed no effects (Supplementary Fig. 2A, B).

## Discussion

Adolescence is a particularly vulnerable time for the onset of psychopathology. Understanding what factors mediate such susceptibility is an important area in biological psychiatry research. In the present studies, we assessed the long-term effects of transient gut microbiota depletion using an established ABX cocktail in mice during adolescence or adulthood. ABX_adolescence_ had long-lasting effects on composition and structure of the gut microbiota and anxiety-like behaviour, whereas ABX_adulthood_ had no effects. This long-lasting shift in the gut microbiota when depleted during adolescence highlights the vulnerability of the gut microbiota during development and may be responsible for the anxiety-like phenotype and pronounced changes in gene expression within the amygdala observed in ABX_adolescence_ mice. To our knowledge, this is the first study demonstrating that the long-lasting effects on gut microbiota only occur when treated during a critical developmental period.

In mammals, the initial microbiota is mainly acquired at birth and later shaped during the developmental period by genetics and environmental factors until become increasingly stable and resistant during adulthood^2,19,21^. Adolescence is a period when major physiological transformation occurs. Sexual hormones such as testosterone and oestrogen are peaking during puberty and it is known to affect the composition of the gut microbiota^2,47,48^. Comparatively, the gut microbiota during the adolescence is less diverse, with a lower Firmicutes/Bacteriodetes ratio than adults^49^. Consequently, environmental stressors such as poor diet, antibiotics, exercise among other can impair the gut microbiota differently in adolescence and adults, with the later demonstrating higher resilience to harmful events compared to adolescence or elderly.

Use of antibiotics strongly affect the Firmicutes/Bacteriodetes ratio^16,18–20,25–27,50–52^ and could be determinant to facilitate changes in brain function and increase susceptibility to anxiety levels^33,49,52^. Evidence has suggested that changes in the microbiota composition can also regulate sexual hormone production, which would have a larger impact during the adolescence^4,47,48^. Thus, disrupting normal growth trajectory of the gut microbiota and its inability to bounce back to its original composition following the treatment may have contributed to the long-lasting effect after ABX treatment in adolescent^53,54^.

Many studies have described that 1–5 weeks of ABX treatment is enough to deplete the gut microbiota and influence the expression of genes and behaviour^8,20,25,26^. By depleting the gut microbiota consistently over most of the adolescence period, we observed a statistically significant increase in anxiety-like behaviour in the EPM only in ABX_adolescence_ mice, in line with the changes in microbiota composition and structure. The role of gut microbiota depletion in anxiety, however, is controversial with studies describing a reduction of anxiety-like behaviours^25,55^, no effect^20,26^ or an anxiolytic effect following microbiota depletion^16^. Firmicutes, Actinobacteria and Bacteroides are known to affect anxiety levels, with lower abundance being associated with elevated anxiety-like behaviour in rodents and humans^36,56–58^. The stronger effect observed in ABX_adolescence_ mice over adults may also have been influenced by sex hormones peaking during adolescence. Preclinical and clinical studies in males suggest that testosterone yields protective benefits against anxiety. Moreover, high levels of testosterone during the adolescent period is known to influence the commensal bacteria Firmicutes/Bacteroides ratio^4,47,48^, suggesting that interaction of ABX with testosterone during adolescence could have an additive effect on the gut microbiota composition.

In addition, it has been found that administration of probiotics containing species of the genus *Lactobacillus* and *Bifidobacterium* correlates with the expression of *Gabrb1* within the amygdala, an effect that has been shown to be influenced by vagal communication^36,59^. Interestingly, *Lactobacilli* species appeared mainly increased and *Bifidobacterium* was unchanged in ABX_adolescence_. Beneficial bacterial species such as *Lactobacillus* and *Bifidobacterium* are usually found decreased after ABX treatment in rodents^60^ and elevated *Lactobacillus*/*Bifidobacterium* levels in the gut microbiota or use of probiotic preparations have an anxiolytic effect in rodents^36,61–63^. These comparisons must be taken carefully since most of the previous studies have not investigated the long-term effects of gut microbiota depletion. Moreover, a study has found that juvenile rodents react differently to treatment with *Lactobacillus*, evoking an anxiogenic-phenotype following by changes in the gene expression and brain function^64^, suggesting that the physiological mediators peaking during the adolescent period may be playing a role on the involvement of the *Lactobacillus* on the gut-brain communication.

It is interesting to note that members of the family *Lachnospiraceae* (in special *NK4A136* group) and *Bacteroides* genus, which were decreased in ABX_adolescence_, are also associated with anxiety in humans^65,66^ as well as relevant to the production of SCFAs in rodents^67^. SCFAs are important bacterial metabolites with neuroactive properties^67,68^. Moreover, colonic SCFAs regulate intestinal barrier function^69,70^. Thus, a dysfunction in the intestinal barrier can increase inflammatory response and facilitate that peripheral substances escape into the bloodstream and potentially reach the brain. Further studies are needed to mechanistically unravel the pathways involved.

Interestingly, despite the body of literature showing that the gut microbiota is implicated in social behaviour or cognitive abilities^17,20,25,26,50,71–74^, we did not observe any overt effect of ABX treatment in social behaviour and short-term memory, suggesting that with the parameters we used these phenotypes were not responsive to long-lasting effects of gut microbiota depletion. Moreover, it has been shown that deficient synaptic pruning, which is associated with weak synaptic transmission and decreased functional brain connectivity, is correlated with deficits in social interaction and other neuropsychiatric disorders^75^. These failures in the synaptic connectivity are due to the lack of activity of the *Cx3cr1* gene^75,76^. *Cx3cr1* was found upregulated in our study, suggesting a local role on brain inflammatory response which is not directly associated with sociability. In addition, fear conditioning which has previously been shown to be modulated by microbiome manipulations^32,36,77^ was only marginally affected by gut microbiota depletion as only ABX_adolecence_ mice showed increased freezing behaviour during fear acquisition, indicating that learning and memory indeed are not overtly influenced by ABX treatment in this circumstances, despite all the changes observed on the gut microbiota composition. To note, increased levels of *Lactobacillus* species are described to improve cognition process in rodents, including learning and memory^61^ but not in a clinical study^78^.

Here we focused on the amygdala to understand to which extent changes in gene expression could underly the differences observed in the anxiety-like behaviour. Despite the highest prevalence of overall gene expression changes in ABX_adolescence_, the administration of ABX induced similar disturbance for genes involved in neuroimmunity response in ABX_adolescence_ and ABX_adulthood_ mice. Given the importance of microglia in normal brain function, it is not surprising that these genes have been associated with the ABX treatment. The microglia-related genes *Fcgr2b* and *Rac2* were upregulated in both groups, while *Cx3cr1* and *C1qa* were elevated only in ABX_adolescence_ mice, confirming a role for microbiota recolonization observed in ABX_adulthood_ (but not in ABX_adolescence_) with restored microglia features^76^. It has been suggested that elevated levels of the *Cx3cr1* ligand CX3CL1 is associated with pro-neurogenic response by altering the environment in which new cells are born^79^, suggesting that together with *C1qa*, *Cx3cr1* are playing a role in the prevention of chronic inflammation induced by the gut microbiota depletion. In fact, microglia seems to keep engulfing apoptotic debris even after antibiotic treatment^80^, which together with elevated *Tlr4* expression followed by ABX_adolescence_ treatment could indicate certain level of neuroinflammation has been occurring without the participation of the cytokines. In addition, it is well known that SCFAs directly affect immune response with the intention to maintain homoeostasis by fine-tuning microglial function and production of inflammatory cytokines, further suggesting that the overexpression of the SCFAs transporters *Slc5a8* and *Slc16a1* in the amygdala of ABX_adolescence_ mice could be related with the microbiome-derived factors that are involved in modulate adaptive immune responses in the brain. *C3* is a downstream member of the component cascade with an extremely versatile role that it is not restricted to immune responses but also in tissue regeneration and synapse pruning^81^. Interestingly, *C3* and *Il1β* were elevated only in ABX_adulthood_. This could suggest that neuronal loss and consequently cognitive deficits associated with long-term depression caused by C3 could have been counteracted by the production of *Il1β*, a cytokine with a neuromodulatory role on neurogenesis^82^.

Tight-junction proteins at the blood-brain barrier are known to be regulated by the gut microbiota, where the decreased expression of these proteins is associated with permeable blood-brain barrier^83,84^. Although gut depletion is necessary to promote tight-junction protein changes in the brain, the direction of these changes is dependent on the brain region and protein tested. Herein, we observed an upregulation of amygdalar *Tjp1* and *Ocln* only in ABX_adolescence_ mice, confirming the role of depleted gut microbiota on brain permeability^26,84^. Moreover, this study also showed that the natural recolonization of the gut microbiota is enough to recalibrate the functionality of the information transfer between the gut and brain following a microbiota-targeted insult. Taken together, our findings show that the immunological markers changed in ABX_adolescence_ and ABX_adulthood_ mice are not a result of a brain leakage through the blood-brain barrier but probably due to local synthesis of volume diffusion into the brain at the circumventricular organs that lack a blood-brain barrier.

When examining the pattern of expression of genes involved in neurotransmission it was clear that they were more strongly affected in ABX_adolescence_ than ABX_adulthood_ mice. Elevated brain levels of *Crh1r* has been found after ABX treatment or in GF mice^6,20^ while downregulation of the transcription levels of *Crh1r* has been found after psychobiotics or SCFAs treatment in stressed animals^67,85^, suggesting that restoration of the normal gut microbiota could explain why we see altered *Crh1r* expression in ABX_adulthood_ mice. In addition, NPY and activation of NPY1R are known to promote stress resilience and have anxiogenic effects^86–89^. NPY locally influences GABAergic activity through NPY1R, and both *Npy* and *Npy1r* were found to be upregulated in chronic stress events^90^, in line with the results observed in ABX_adolescence_ mice. In fact, evidence suggests that the gut microbiota may contribute to resilience after repeated stress^91–93^. For instance, it has been shown that chronic stress significantly increases gut microbiota diversity in antibiotic-treated mice and can neutralize not only stress-inducing anhedonia phenotypes but also the levels of inflammatory markers, suggesting that the development of susceptibility to stress in mice is subjected to the composition of the gut microbiota^93^. Recently, evidence has also confirmed that the use of commensal bacteria strain to improve psychological functions and cognitive health in stressed adult subjects^58^.

Interestingly, genes related with neuroplasticity, postsynaptic density protein 95 (*Psd95*) and synaptophysin (*Syp*) were oppositely affected by ABX_adolescence_ and ABX_adulthood_, suggesting a direct role of the gut microbiota composition on the expression of these genes. A recent study has shown that at least in the PFC, ABX treatment protects *Psd95* expression from stress, suggesting that depleted gut microbiota could play a role in the development of susceptibility in mice after stress^93^. However, in this study, stress started after the ABX treatment, thus investigating the role of stress during the recovery period of the gut microbiota, in agreement with previous literature that found normalized levels of *Syp* and *Psd95* after the restoration of the gut microbiota^9^. Therefore, the upregulated expression of these genes in ABX_adolescence_ mice could be due to the long-lasting shift in microbiota composition and structure, whereas the decrease observed in ABX_adulthood_ mice might come from an exaggerated downregulation of these genes due to a possible overexpression during the gut microbiota depletion period.

Unexpectedly, ABX_adulthood_ treatment seems also to play a role in myelin-related gene. Recent studies have shown upregulation of myelination-related genes in the PFC of animals with disrupted gut microbiota, suggesting a role for the gut microbiota in the formation of myelin^37^. Our study indicates that depletion of the gut microbiota does not exclusively affect myelination during critical developmental periods but can also influence myelination-related gene expression in adulthood. Whether these changes in gene expression translates to altered myelination, however, still needs to be investigated.

Overall, this study highlights vulnerability of the gut microbiota during the adolescent period and the importance of the microbiota during the developmental period in shaping its host’s microbiota, brain function and behaviour. A limitation of this study is that only males were tested. It is worth pointing out that for the majority of our studies of microbiota to brain interactions the effects are much more robust in males^94^. However, much research is still needed to unravel this interaction, and recent evidence suggests that besides the sex bias for mental disorders, an interaction between the sex, sex hormones and the gut microbiota is highly possible with the gut microbiota^94^. Future studies will determine how sex and sex hormones determine discrete genes changes in the amygdala as well in the gut. Further, understanding what pathways of communication between the gut and the brain are responsible for such changes at this time period are also required. Interventions targeting the vagus nerve or the use of specific dietary interventions focused on specific populations of commensal bacteria that are affected by ABX would be of further interest to provide a mechanistic framework of the gut to brain signalling^29,95^. Finally, as adolescence is a time where antibiotics are heavily used clinically^28^, and its use is epidemiologically relevant to the risk of several psychiatric conditions^96^, the results presented here support the need for further studies investigating their impact and their long-term effects on the microbiota and associated risks to the brain function and behaviour.

## Supplementary information

Supplemental Material
