# Supplementary material for: Enduring neurobehavioral effects induced by microbiota depletion during the adolescent period
Source: Transl Psychiatry. 2020 Nov 6;10:382. doi: 10.1038/s41398-020-01073-0 (PMC7648059; doi:10.1038/s41398-020-01073-0)
Supplement: Supplementary file 1 — Supplemental Material [file 41398_2020_1073_MOESM1_ESM.doc]

**Supplementary Information**

**Supplementary Methods**

**Elevated Plus maze**

The EPM is a commonly used paradigm to investigate anxiety-like behaviours in rodents. Mice were habituated to the testing room for 60 minutes prior to testing. The maze was elevated 60 cm from the floor, comprising of two open (30 x 5 cm, W x L) and closed arms (30 x 5 x 15 cm W x L x H). Grip on the edge of the open arms was facilitated by inclusion of a small, raised edge (0.25 cm). All arms of the maze were cleaned with 70% ethanol before introduction of the animal. Facing the open arm, each animal was placed into the centre platform and allowed to explore for 5 minutes. The procedure was carried out under red light (5 lux on open arms). Test sessions were recorded by a video camera positioned directly above the apparatus. The time spent in each arm, the number of entries to the open and closed arm and the number of head dip were manually scored by an observer blind to the experiment.

**Novel object recognition (NOR)**

Mice were habituated to the setup 24h hours before the test. Mice were tested under 60 lux in a grey plastic box (40 cm x 30 cm x 25 cm, L x W x H). One hour after investigation of two identical objects, mice are presented with an old and a new object. The time mice spent with either of the objects gives an indication on memory function. Test sessions were recorded by a video camera positioned directly above the apparatus and the time of direct contact with the objects were scored manually by an observer blind to the experiment.

**Three-chamber social approach task**

The test was run in a rectangular apparatus (36 x 19 x 30 cm, L x W x H) divided into 3 inter-connected chambers. The test procedure was comprised of three 10 min exploration trials, each starting from the middle chamber. 1) Habituation trial: the arena had two empty mesh wire cages symmetrically positioned in the side chambers. 2) “Mouse vs. Object” trial: an object and an age-, sex- and strain-matched unfamiliar mouse was placed into one of the wire cages in either the left or right-side chamber. 3) “Novel vs. Familiar Mouse”: an object was replaced with an unfamiliar mouse. The test was conducted at 60 lux in the middle chamber. Behaviours were video recorded. Preference in exploration shown by the test mouse was assessed in % according to the formulas: t [mouse] / (t [mouse] + t [object]) and t [novel mouse] / (t [novel mouse] + t [familiar mouse]).

**Differential fear-conditioning paradigm**.

This test is based on the learning of the association of an initially neutral and non-aversive stimulus, a tone cue or context (conditional stimulus, CS), with an aversive one, such as foot shock (unconditional stimulus, US). Fear is measured by the freezing response (absence of movement except for breathing) in response to the presentation of the neutral stimulus cue or context (CS). The protocol used was based on a paradigm combining context and cue, allowing to differentiate the behavioural responses to each of these components. The experiment was performed in two different contexts, named context A (day 1 and 2) and context B (day 3 and 4). Context A consisted of a transparent acrylic rodent conditioning chamber with a metal grid floor that was enclosed by a sound attenuating chamber. Illumination was 80 lux and chambers were cleaned with 70% ethanol after each session. Fear extinction and recall were performed in context B consisting of a dimly illuminated (10 lux) chamber with black, smooth, walls with a 30° angle and cleaned with 1% acetic acid. On day 1 mice were subjected to a differential fear-conditioning paradigm during which an auditory stimulus served as a conditioned stimulus (CS+, 30 s white noise, 80 dB) as it was paired with a US. All animals received 5 CS+ (random inter-stimulus interval) starting 120s after the mice had been placed in the chamber. The unconditioned stimulus (US) co-terminating with each CS+ consisted of a mild electric foot shock. The shock intensity was set to 0.5 mA (2 s). Mice were removed from the apparatus 120s after the last foot shock. On day 2, mice were exposed to context A for 12 min to measure contextual fear. Fear extinction was performed on day 3 as followed: after a 2 min habituation period, 25 presentations of CS+ (30 s, inter-stimulus interval 5 s) was performed, and mice taken out of the box 2 min after the last CS presentation. Extinction recall was tested on day 4 by presenting 5 CS+ (30 s, inter-stimulus interval 5 s) in context B. Freezing was assessed by automated video freezing tracking software (Video Freeze, Med Associates Inc, USA). Percentage freezing during tone presentation was determined. For determination of percentage freezing using the Video Freeze software a motion threshold index (au) of 18 was used and min freeze duration (f) of 30. Freezing during extinction was averaged into 5-trail blocks for analysis.

**Supplementary figures, tables and legends**

**Figure S1**


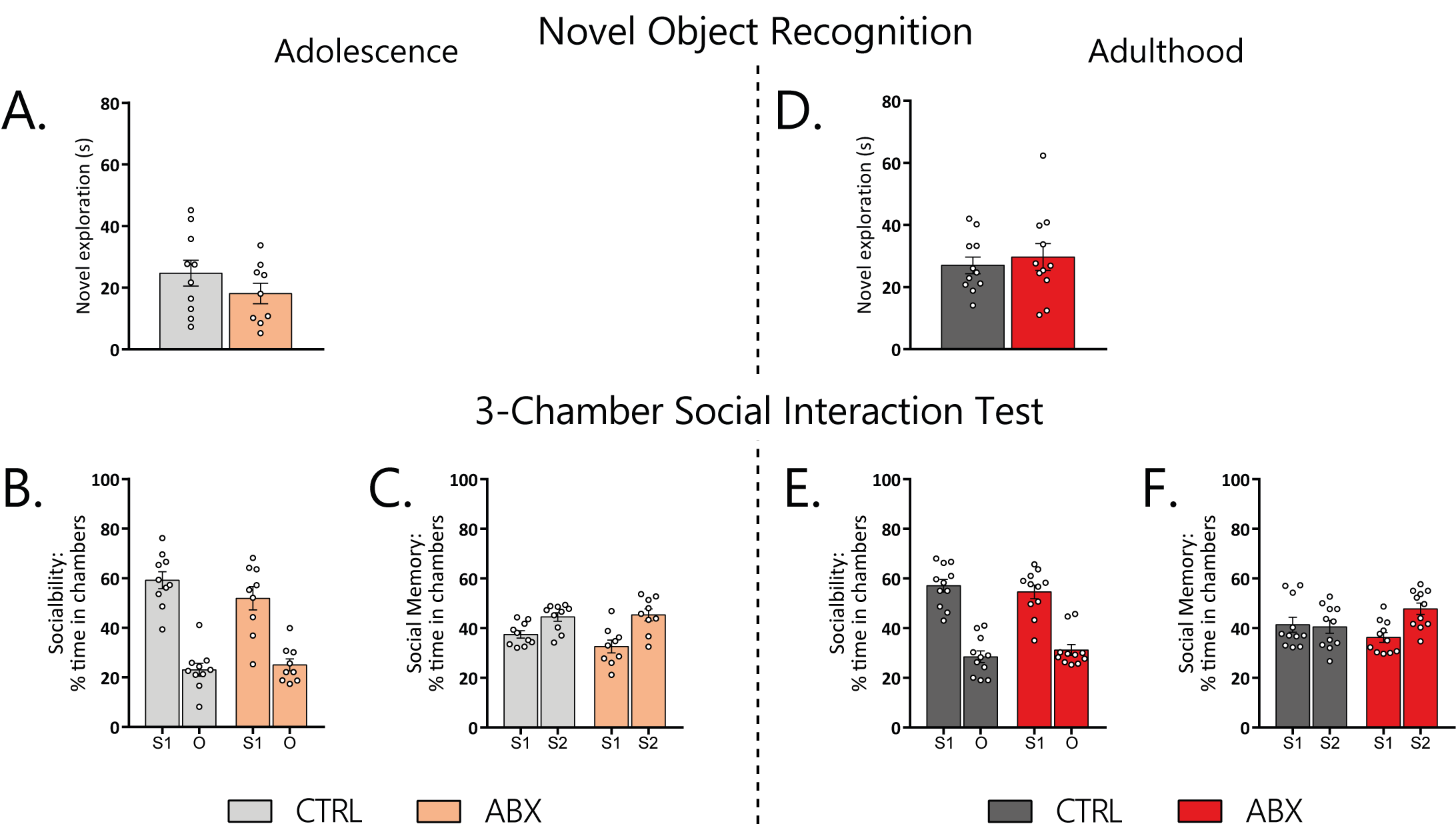


**Legend Figure S1: ABX treatment does not affect working memory, sociability or social memory. A, D** No difference is observed in the time mice spent exploring the novel object ABX_adolescence_ and ABX_adulthood_ mice when compared to their respective controls. **B, E** Sociability was also not affected by ABX_adolescence_ or ABX_adulthood_ treatment. All groups showed a significant preference for the mouse (S1) over an innate object (O). **C, F** ABX_adolescence_ or ABX_adulthood_ but not their controls showed a preference investigating the novel mouse (S2) over a familiar mouse (S1). Mean ± SEM. **A, D** Welsh’s t test comparison between treatments. **B, C, E, F** Two-way repeated measures ANOVA followed by Sidak’s post hoc test ***p<0.001. Sample size for Adolescence (CTRL: n=9; ABX: n=10) and Adulthood (CTRL: n=11; ABX: n=11).

**Figure S2**


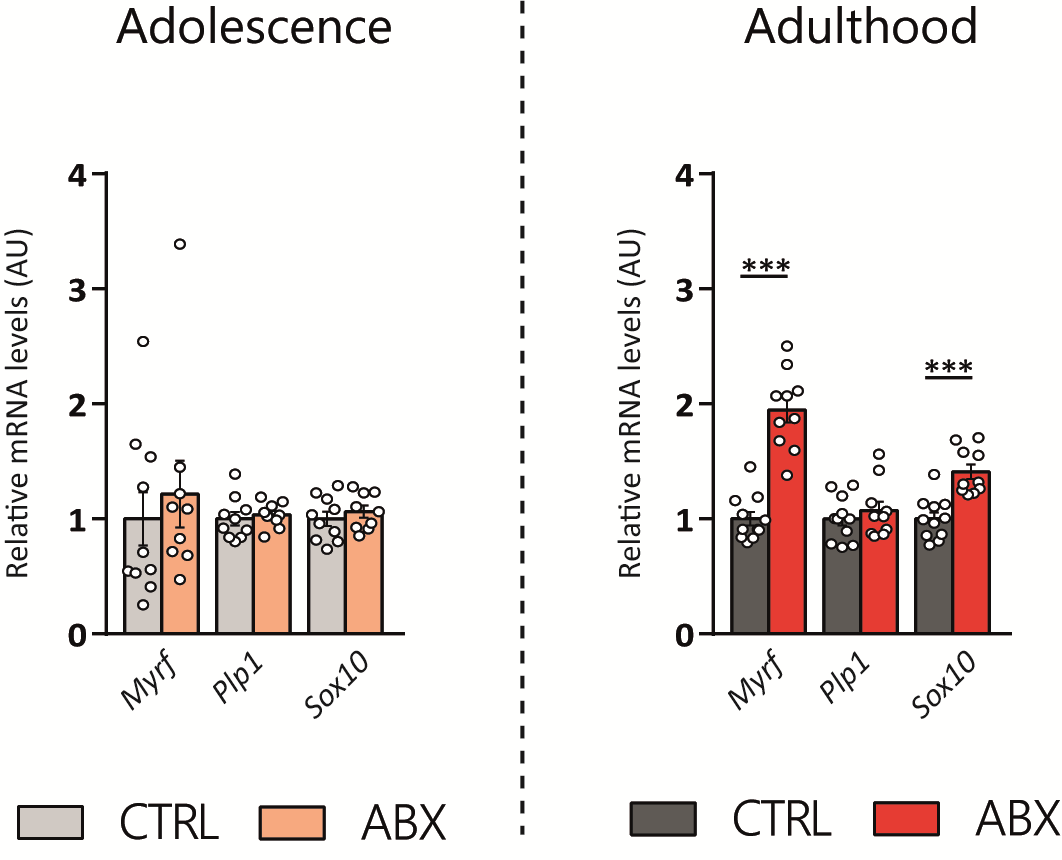


**Legend Figure S2: Altered myelination-related gene expression was observed in the Prefrontal Cortex of mice treated with ABX during adulthood. A, B** MYRF and SOX10 were significantly upregulated in ABX_adulthood_ mice, but myelination-related gene expression was not affected in ABX_adolescence_ mice. Mean ± SEM. Unpaired t-test with Welch's correction comparing CTRL and ABX mice for each gene. ***p<0.001. Sample size for Adolescence (CTRL n=10 and ABX n=9) and adults (CTRL n=11 and ABX n=10), adult (n=10-11). *Myrf*: Myelin Regulatory Factor, *Plp1*: Proteolipid Protein 1, *Sox10*: SRY-Box Transcription Factor 10.

**Table S1: List of sequencing primers**. *C1qa*: complement C1q subunit A, *C3*: complement component 3, *Crh1r*: corticotropin-releasing hormone receptor 1, *Cx3cr1*: chemokine receptor 1, *Fcgr2b*: Fc Fragment Of IgG Receptor IIb, *Ffar1*: free fatty acid receptor 1, *Gabrb1*: gamma-aminobutyric acid type B receptor subunit 1, *Gabra2*: gamma-aminobutyric acid type A receptor alpha2 subunit, *Grm5*: glutamate metabotropic receptor 5, *Il1b*: Interleukin 1b, *Il10* interleukin 10, *Myrf*: Myelin Regulatory Factor, *Npy*: neuropeptide Y, *Npy1r*: neuropeptide Y receptor Y1, *Nr3c1*: glucocorticoid receptor, *Ocln*: occludin, *Plp1*: Proteolipid Protein 1, *Psd95*: postsynaptic density protein 95, *Rac2*: Ras-related C3 botulinum toxin substrate 2, *Slc5a8*: Solute Carrier Family 5 Member 8*,* *Slc16a1*: Solute Carrier Family 16 Member 1, *Sox10*: SRY-Box Transcription Factor 10, *Syp*: Synaptophysin, *Tjp1*: tight junction protein 1, *Tlr4*: toll-like receptor 4.

**Table S2: Statistical data for each individual DNA sequences recovered from high-throughput analysis of the gut microbiota.** ASV: Amplicon sequence variant.
